# Management and Outcomes in the Elderly with Non-ST-Elevation Acute Coronary Syndromes Admitted to Spoke Hospitals with No Catheterization Laboratory Facility

**DOI:** 10.3390/jcm11206179

**Published:** 2022-10-20

**Authors:** Francesca Mantovani, Gianluca Campo, Elisa Guerri, Francesco Manca, Massimo Calzolari, Giovanni Tortorella, Sergio Musto D’Amore, Gianluca Pignatelli, Vincenzo Guiducci, Alessandro Navazio

**Affiliations:** 1 Cardiology Unit, Azienda USL-IRCCS di Reggio Emilia, Viale Risorgimento 80, 42123 Reggio Emilia, Italy; 2 Cardiology Unit, Cardiovascular Institute, Translational Medicine Department, University of Ferrara, 44121 Ferrara, Italy; 3 Cardiology Unit, AUSL di Parma, Ospedale Vaio-Fidenza, 43036 Vaio, Italy

**Keywords:** elderly, service strategy, coronary artery angiography, acute coronary syndrome, spoke hospital, same-day transfer

## Abstract

Background: Contemporary guidelines advocate for early invasive strategy with coronary angiography in patients with non-ST-elevation acute coronary syndromes (NSTE-ACS). Still, the impact of an invasive strategy in older patients remains controversial and may be challenging in spoke hospitals with no catheterization laboratory (cath-lab) facility. Purpose: The purpose of this study was to analyse the characteristics and outcomes of patients ≥80 years old with NSTE-ACS admitted to spoke hospitals. Methods: Observational–retrospective study of all consecutive NSTE-ACS patients admitted to two spoke hospitals of our cardiology network, where a service strategy (same-day transfer between a spoke hospital and a hub centre with a cath-lab facility in order to perform coronary angiography) was available. Patients were followed up for 1 year after the admission date. Results: From 2013 to 2017, 639 patients were admitted for NSTE-ACS; of these, 181 (28%) were ≥80 years old (median 84, IQR 82–89) and represented the study cohort. When the invasive strategy was chosen (in 105 patients, or 58%), 98 patients (93%) were initially managed with a service strategy, whereas the remainder of the patients were transferred from the spoke hospital to the hub centre where they completed their hospital stay. Of the patients managed with the service strategy, a shift of strategy after the invasive procedure was necessary for 10 (10%). These patients remained in the hub centre, while the rest of the patients were sent back to the spoke hospitals, with no adverse events observed during the back transfer. The median time to access the cath-lab was 50 h (IQR 25–87), with 73 patients (70%) reaching the invasive procedure <72 h from hospital admission. A conservative strategy was associated with: older age, known CAD, clinical presentation with symptoms of LV dysfunction, lower EF, renal failure, higher GRACE score, presence of PAD and atrial fibrillation (all *p* < 0.03). At the 1-year follow-up, the overall survival was significantly higher in patients treated with an invasive strategy compared to patients managed conservatively (94% ± 2 vs. 54% ± 6, *p* < 0.001; HR: 10.4 [4.7–27.5] *p* < 0.001), even after adjustment for age, serum creatinine, known previous CAD and EF (adjusted HR: 2.0 [1.0–4.0]; *p* < 0.001). Conclusions: An invasive strategy may confer a survival benefit in the elderly with NSTE-ACS. The same-day transfer between a spoke hospital and a hub centre with a cath-lab facility (service strategy) is safe and may grant access to the cath-lab in a timely fashion, even for the elderly.

## 1. Background

Recent evidence has shown the advantages of an early invasive strategy (coronary angiography ± percutaneous coronary intervention (PCI)) for patients with intermediate–high-risk non-ST-segment elevation acute coronary syndrome (NSTEACS) admitted to a hospital; such strategy is now recommended in the international guidelines [1]. However, these recommendations are based on large randomized trials with a mean age of participants of ~65 years and, since few patients in their 80s were enrolled, the survival benefit cannot be presupposed to translate to these patients [2]. In routine clinical practice, frail patients with several comorbidities are more likely to be treated non-invasively, whereas the fittest patients are more likely to undergo invasive management. Therefore, the rate of invasive coronary angiography declines with age; only 38% of patients with NSTEMI who are older than 80 years receive a coronary angiogram, compared with 78% of those aged 60 years or younger [3]. Moreover, practice gaps in patients’ management have been noticed between hospitals with or without a catheterization laboratory (cath-lab) facility (hub centres vs. spoke centres) [4,5,6,7,8]. The service strategy is explained as the same-day transfer between the referring non-invasive spoke hospital and the hub centre with cath-lab facility; it has been shown to be a safe option and to minimise these inconsistencies [9,10,11,12,13,14]. The aim of this study was to retrospectively analyse the implementation of the service strategy and the 1-year outcomes of the elderly admitted in spoke hospitals of an Italian cardiology network.

## 2. Methods

### 2.1. Study Population and Data Collection

We retrospectively enrolled all consecutive patients aged ≥80 years [15], admitted to two spoke hospitals of our cardiology network in Reggio (Guastalla and Castelnovo nè Monti) and diagnosed with NSTEACS from January 2013 to December 2017.

NSTEACS patients were detected in data retrieved from hospital administrative systems using discharge diagnoses codes for non-ST-segment elevation myocardial infarction (NSTEMI) and unstable angina (UA) (International Classification of Diseases, Ninth Revision, Classification Modification, or ICD-9, codes: 410.7, 411.1, 411.81 and 411.89). The study population was divided into two groups according to the management chosen: invasive vs. conservative. 

Hospital notes were examined for more information, whenever they were judged to be needed. All-cause death was the endpoint of the study. Follow-up information for death was acquired from the national death index, where the status of all citizens is securely and constantly updated and is 100% complete. Indeed, in Italy, it is mandatory by law that all deceased patients are instantly recorded in this national data bank. Since the present retrospective analysis did not alter the contemporary clinical practice in the reported institutions, the regulatory authorities did not require any supplementary written informed consent for data gathering. The ordinary written consent for coronary angiography and data privacy was acquired from all patients.

### 2.2. The Cardiology Network 

**Figure 1:**
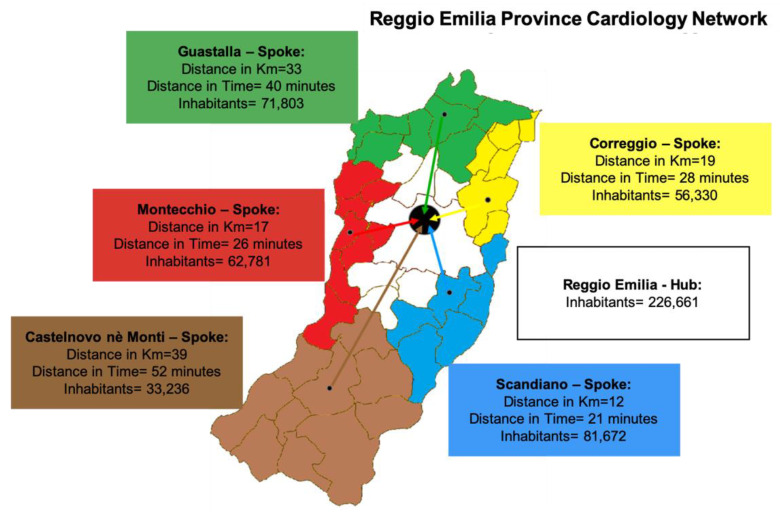
The cardiology network in Reggio Emilia. Six hospitals serve the Province of Reggio Emilia. Hospitals are organized according to their facilities: one invasive hub hospital with catheterisation laboratory (cath-lab) with 24/7 service (Reggio Emilia) and five non-invasive spoke hospitals (Guastalla and Castelnovo nè Monti, whose data are the object of the present study and Correggio, Scandiano, Montecchio). The distance between the spokes and the hub centres are expressed in km and time.

Reggio Emilia is an area of the Italian Emilia-Romagna region with nearly 532,000 inhabitants. Hospitals are organized according to their facilities as follows: (a) one hub hospital with intensive cardiac care unit (ICCU) and catheterisation laboratory (cath-lab) with 24/7 service for primary PCI (Reggio Emilia); (b) two spoke hospitals with ICCU (Guastalla and Castelnovo nè Monti), whose data are object of the present study; (c) three spoke hospitals with internal medicine departments and cardiology consultation services without ICCU (Correggio, Scandiano, Montecchio) Figure 1.

Patients with NSTEACS, triaged by the emergency medical system or self-presenting, were generally admitted to the nearest hospital with ICCU, regardless of the presence of a catheterization laboratory. Only patients with NSTEACS and haemodynamic-instability criteria were sent straight to the hub centre. In the spoke hospitals, the NSTEACS patients’ appropriateness for coronary angiography ± PCI was at the discretion of the clinician, after a stratification of ischaemic and bleeding risk with the GRACE score, and consideration of comorbidities and clinical status. If coronary angiography was suggested, the hub centre would be contacted to plan the procedure. According to the clinical case, patients could be managed with service strategy (see below definition), or with a strategy of transfer from the spoke hospital to the hub centre, where patients underwent coronary angiography ± PCI and concluded their hospital stay with no return to the spoke centre.

### 2.3. Service Strategy: Description 

The service strategy has been formerly illustrated in detail in [11,13,14].

To briefly illustrate this strategy, the NSTEACS patients hospitalised in spoke hospitals were transferred to the cath-lab of the hub centre the day of coronary angiography. Once there, the interventional cardiologist revised the medical record and re-evaluated the suitability for the procedure. Then, coronary angiographies ± PCI were performed. All invasive procedures were performed according to the standard interventional technique. Medical therapy (including heparin, P2Y12 receptor inhibitors, aspirin, glycoprotein IIb/IIIa inhibitors and bivalirudin) was ordered according to contemporary guidelines [1].

After the coronary angiography, patients were monitored in the cath-lab recovery room for nearly 4–5 h. After this time (and within the same day), patients were sent back to the referring spoke centre, attended by basic-life-support-certified staff.

Severe angiographic complications, hemodynamic instability or logistical motivations were considered as reasons preventing the return to the referral centre.

### 2.4. Endpoint

The principal objective of the present study was to describe: (a) characteristics; (b) management and strategy chosen; (c) time between hospital admission to spoke centres and cath-lab access; and (d) outcomes in terms of all-cause death of patients ≥80 years old with NSTE-ACS admitted to spoke hospitals.

## 3. Statistical Analysis 

Continuous variables were expressed as mean ± standard deviation (SD) or mean and interquartile range (IQR) and were compared by the unpaired *t*-test. Categorical variables were expressed as counts and percentages and the comparison was performed by the chi-square test. 

Kaplan–Meier curves were constructed to show survival according to strategy chosen (invasive or conservative). Univariate and multivariable Cox proportional hazards models were used to assess the association between strategy and the risk of death; the risk was presented as hazard ratio (HR) and 95% confidence interval (CI).

The level of statistical significance was set at 5% (*p* < 0.05) and all statistical analyses were carried out using SPSS (version 15 for Windows).

## 4. Results 

### 4.1. Demographic and Clinical Characteristics of the Study Population 

Table 1 shows the main baseline characteristics of the study population. 

From January 2013 to December 2017, 639 consecutive patients were admitted to spoke centres with a diagnosis of NSTEACS. Of these, 181 (28%) were ≥80 years old and represented the study cohort. The median age of the study population was 84 (IQR 82–89) years old. The most frequent clinical presentation was non-ST elevation myocardial infarction in 145 patients (80%); the remainder of the patients presented with unstable angina. Forty-six patients (25%) showed clinical signs of left-ventricular dysfunction and one hundred fifty-one patients (83%) were in sinus rhythm at admission. The proportion of cardiovascular risk factors in the study population comprised: smoke habit in 39%, dyslipidaemia in 50%, diabetes in 23%, arterial hypertension in 73% and known previous CAD in 45%. Major comorbidities in the study population showed the presence of severe chronic kidney disease requiring dialyses in 2%, severe chronic obstructive pulmonary disease (COPD) in 11% and peripheral-artery disease (PAD) in 32%.

Left-ventricle ejection fraction (LVEF) was 47 ± 12%. Serum creatinine was 1.2 ± 0.6 mg/dL.

The calculated GRACE score was 176 ± 29.

**Figure 2:**
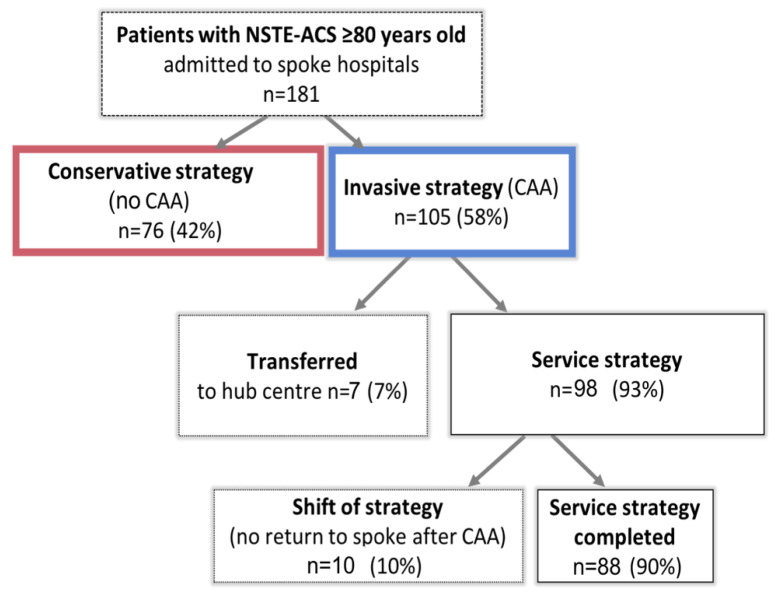
Study population according to the chosen management of NSTE-ACS (non-ST-elevation acute coronary syndrome); CAA (coronary artery angiography).

The study cohort was divided into two groups according to management: invasive strategy in 105 (58%) while the remainder were managed with the conservative strategy. Figure 2.

### 4.2. Management Strategy and Time between Spoke Admission and Access to the Cath-Lab

When the invasive strategy was chosen, 98 patients (93%) were initially managed with a service strategy, whereas the rest of the patients (7%) were transferred from the spoke hospital to the hub centre and completed their hospital stay without returning to the spoke centre for clinical or organizational reasons. Of the patients initially managed with the service strategy, a shift of strategy was necessary after the invasive procedure for 10 (10%) and the patients remained in the hub centre until discharged home with no return to the spoke hospital, mainly for clinical reasons; the rest of patients were sent back to the spoke hospitals, with no adverse events observed during the transfer. Figure 2. The median time for access to the cath-lab was 50 h (IQR 25–87), with 73 patients (70%) reaching the invasive procedure <72 h from hospital admission and 23 patients (22%) reaching the invasive procedure in <24 h. The mean hospital-stay length was 6 ± 3 days (median value 5 days; IR 4–8).

### 4.3. Conservative vs. Invasive Strategy

In the elderly, the conservative strategy was chosen in 76 patients (42%). Conservative strategy was found to be associated with older age, smallest body mass index, higher prevalence of known CAD, clinical presentation with symptoms of LV dysfunction, lower EF, worse renal failure, higher GRACE score, higher prevalence of PAD and atrial fibrillation (all *p* < 0.03). The choice of strategy did not affect the length of hospital stay (*p* = 0.37).

**Figure 3:**
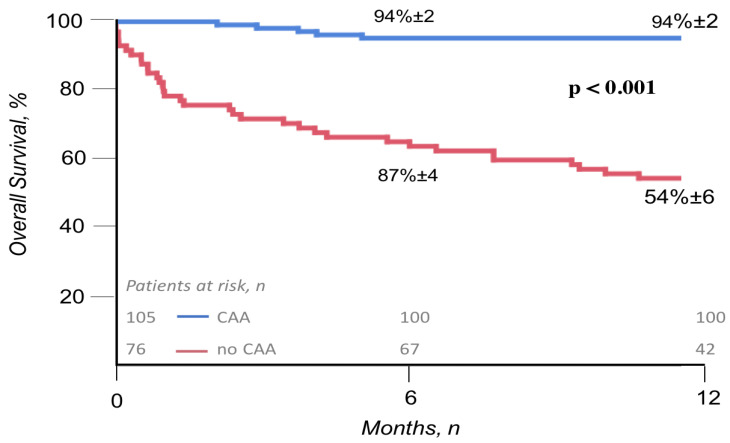
Overall 1-year survival after NSTEACS in patients treated with invasive strategy (blue line) and conservative strategy (red line). Figure legend: CAA (coronary artery angiography).

At 1-year follow-up, the overall survival was significantly higher in patients treated with the invasive strategy compared to patients managed conservatively (94% ± 2 vs. 54% ± 6, *p* < 0.001; HR: 10.4 [4.7–27.5] *p* < 0.001), even after adjustment for age, serum creatinine, known previous CAD and EF (adjusted HR: 2.0 [1.0–4.0]; *p* < 0.001). Figure 3.

### 4.4. Discussion 

Our observational–retrospective study showed that, in our provincial cardiology network, the treatment of elderly people admitted to spoke hospitals with NSTEACS was characterised as follows: (a) they were managed invasively in more than half of cases; (b) the invasive strategy conferred a 1-year survival benefit compared to the conservative strategy; (c) the service strategy represented an effective and safe strategy to ensure access to the cath-lab in a timely fashion (<72 h) in the vast majority of elderly patients (70%).

The elderly (aged 80 or older) characterize a growing proportion of the patients presenting with NSTEMI, but these patients are much less likely to receive invasive management. 

Data from the National Inpatient Sample database in the USA indicated that 78% of patients with NSTEMI aged 60 years or younger underwent coronary angiography, compared with 38% of patients aged 81 years or older [3]. In the SENIOR-NSTEMI study, 49% of eligible patients from five tertiary centres in UK underwent invasive management during their index admission [16]. 

In our study, 58% of patients older than 80 years underwent coronary angiography.

These differences in treatment strategy between elderly and younger patients likely reflect insufficient data to guide clinical practice. In fact, recommendations are based on large randomized trials with a mean age of participants of ~65 years and, since few patients in their 80s were enrolled, the survival benefit cannot be assumed to translate to these patients [2]. Treatment decisions are habitually made in the elderly within the clinical context of a delicate evaluation of risks and benefits according to comorbidities and estimated life expectancy. Particularly, clinicians fear bleeding risk and the risk of acute kidney injury in decision making regarding the decision around invasive management in the elderly. In a sub-study of the Randomized ANTARCTIC Trial, clinically relevant bleeding events were observed in 20% of elderly patients undergoing percutaneous coronary angiography for an ACS and were strongly associated with further stroke occurrence [17]. Rather than the antiplatelet therapy, comorbidities and an age > 85 years predicted bleeding outcomes in this elderly population [17]. Moreover, the elderly are at incremented risk of acute kidney injury due to several aging-related factors, such as nephrosclerosis, inflammation and vascular changes [18,19]. Between older adults undergoing cardiac catheterization for acute myocardial infarction in the SILVER-AMI study, nearly one in five experienced acute kidney injury [20]. Development of acute kidney injury after coronary angiography is associated with worse outcomes [21], including increased length of stay, excess costs, progression to end-stage renal disease and mortality, with predictors that largely mirrored those described in previous studies of younger patients [20]. 

In our study, patients managed with the conservative strategy were associated with: older age; smallest body mass index; higher prevalence of known CAD; known complex coronary lesions in most cases being previously judged to not be treatable by percutaneous coronary intervention; clinical presentation with symptoms of LV dysfunction; lower EF; worse renal failure; higher GRACE score; higher prevalence of PAD (possibly complicating in the choice of arterial accesses) and atrial fibrillation (raising concerns about bleeding risk on triple-anticoagulant therapy); and having worse prognosis at 1-year follow-up. 

Despite international guidelines continuing to recommend that older patients be considered for invasive management and revascularization (class IIa recommendation) [1,22,23], these recommendations are based on small randomised trials (Italian Elderly ACS [24] (invasive group *n* = 154 and non-invasive group *n* = 159) and the After Eighty trial [15] (invasive group *n* = 229 and non-invasive group *n* = 228)) and small post hoc subgroup analyses of randomised trials (TACTICS-TIMI 18 [25] and FIR [26]) that have evaluated invasive management versus non-invasive management for NSTEMI in patients aged 75–80 years or older. Moreover, a meta-analysis pooling of these data did not find evidence that invasive management reduced mortality at long-term follow-up [27]. In the SENIOR-NSTEMI study, the adjusted cumulative 5-year mortality was 36% in the invasive management group vs. 55% in the non-invasive management group (adjusted hazard ratio 0.68, 95% CI 0.55–0.84). Therefore, the survival advantage of invasive compared with non-invasive management appeared to extend to patients with NSTEMI who are aged 80 years or older [16]. The ongoing SENIOR-RITA trial aims to randomly assign 1668 patients with NSTEMI aged 75 years or older to receive invasive or non-invasive management. The primary outcome is a composite of cardiovascular death and non-fatal myocardial infarction, and the planned follow-up is 5 years; the study is estimated to be completed in 2024 [28].

In absence of convincing evidence from randomised trials, a few studies from registries [29,30,31] have indicated a benefit from invasive therapy, but the findings might have been amplified by immortal time bias [32] and the inclusion of very frail patients who were certain to be managed non-invasively [33]. Studies of temporal trends from registry data in the US and Europe suggest that, over the past two decades, the progressive switch from a non-invasive to a more invasive approach in older patients with NSTEMI has been accompanied by declining mortality [3,34,35]. 

Moreover, the admission to a tertiary (hub) centre with a cath-lab facility or to a spoke centre without a cath-lab facility might make a difference in treatment choice, especially for the elderly.

Several studies have described a distinct scenario in spoke centres with no cath-lab facilities compared to tertiary centres [6,7,8,36]. In the Italian BLITZ 2 registry, just 36% of patients admitted to spoke hospitals were managed with an invasive strategy [36]. The restricted number of available beds in hub centres with a cath-lab has been indicated as potential cause of this discrepancy between centres. In addition, in NSTEACS patients, the correct timing for coronary angiography plays a central role [22,23]. Guidelines suggested an invasive strategy during the same hospital stay and, if possible, within 72 h from admission [1,23]; this timeframe has recently been further lowered to <24 h [22]. 

While the number of patients with NSTE-ACS needing early coronary angiography is expected to grow, the number of available beds in hub hospitals may not increase accordingly.

Consequently, a possible solution to these discrepancies is to establish fast-track lines for patients in need of coronary angiography ± PCI treatments. Therefore, a healthcare model based on service strategy (patients’ same-day transfer back to the spoke-referring hospital after invasive procedure) has been judged to solve the bed-shortage at hub centres. After coronary angiography and ad hoc PCI, previous studies showed that patients with NSTEACS might be safely re-transferred to the spoke hospital after a few hours of observation [11,13,14,37]. Our previous study on ~1000 patients with NSTEACS managed with a service strategy confirmed that the adoption of this strategy in our province network is safe and allowed access to coronary angiography in a timely fashion [38]. Traditionally, in our regional network, the percentage of patients with NSTEACS referred for coronary angiography from spoke to hub centres was relatively high (73%, 95% CI 71.5–74.5%) and a service strategy was significantly associated with early access to the cath-lab and with a consequent reduction in the hospital stay length [37]. The present study confirms the available evidence even in the subgroup of patients aged older than 80 years.

## 5. Limits of the Study 

Limitations should be taken into account in the interpretation of the present data. 

The main limitations of our study relate to its observational–retrospective nature and its small sample size. In addition, we did not have information on whether there was a differential receipt of evidence-based cardiac care in the non-invasive management group, including prescription of medications. 

Moreover, we acknowledge that the elderly patients managed with the conservative strategy may have several confounding factors which might be associated with the worst outcomes (cancer, severe frailty, high bleeding risk, etc.) compared to patients managed with the invasive strategy. These factors have not been fully adjusted with multivariate analysis. 

However, thus far, there are no data in the literature on management and outcomes on elderly NSTEACS patients admitted to spoke hospitals. Therefore, we believe our study may be of interest despite its limitations.

## 6. Conclusions

This study provides supporting evidence for an invasive approach for treatment of elderly people with NSTEACS.

However, this group of patients is still undertreated, especially when admitted to spoke centres with no cath-lab facilities. A well-organised network with a service strategy for early access to coronary angiography is safe and could guarantee access to cath-labs, even for patients older than 80 years with NSTEACS who have been admitted to the spoke hospitals.

## Figures and Tables

**Table 1:** Demographic and clinical characteristics of the study population.

Table	Invasive Strategy, *n* = 105 (58%)	Conservative Strategy *n* = 76 (42%)	Total *n* = 181	*p* Value
Age, years	84 ± 3	88 ± 5	86 ± 4	<0.001
Male sex, *n* (%)	51 (49%)	43 (57%)	94 (52%)	0.28
Weight, kg	72 ± 12	63 ± 13	70 ± 12	0.005
BMI	26 ± 4	24 ± 4	26 ± 4	0.02
Smoke habit, *n* (%)	4 (4%)	2 (3%)	6 (3%)	0.1
Dyslipidaemia, *n* (%)	68 (65%)	23 (30%)	91 (50%)	<0.001
Diabetes, *n* (%)	25 (24%)	19 (25%)	44 (23%)	0.48
Hypertension, *n* (%)	79 (75%)	54 (71%)	133 (73%)	0.44
Known CAD, *n* (%)	38 (36%)	44 (56%)	82 (45%)	0.009
Clinical presentation: -Unstable angina, *n* (%) -NSTEMI, *n* (%)	25 (24%) 80 (76%)	11 (14%) 65 (86%)	36 (20%) 145 (80%)	0.12
Clinical presentation: -symptoms of LV dysfunction, *n* (%)	9 (9%)	37 (49%)	46 (25%)	<0.001
Serum creatinine, mg/dL	1.1 ± 0.5	1.5 ± 0.8	1.2 ± 0.6	<0.001
Chronic renal failure requiring dialysis	1 (1%)	3 (4%)	4 (2%)	<0.001
EF, %	50 ± 11	44 ± 13	47 ± 12	0.002
GRACE score	170 ± 26	186 ± 33	176 ± 29	0.001
Severe COPD, *n* (%)	8 (8%)	11 (14%)	19 (11%)	0.13
PAD, *n* (%)	22 (21%)	37 (49%)	59 (32%)	<0.001
Atrial fibrillation/flutter, *n* (%)	12 (11%)	18 (47%)	30 (17%)	0.004
Length of hospital stay, days	6.0 ± 3.5	6.5 ± 3.7	6.5 ± 3.6	0.37

Table legend: BMI (body mass index); CAD (coronary artery disease); NSTEMI (non-ST elevation myocardial infarction); LV (left ventricle); EF (ejection fraction); COPD (chronic obstructive pulmonary disease); PAD (peripheral-artery disease).

## Data Availability

Not applicable.
